# The New Amendment Act

**Published:** 1915-03-27

**Authors:** 


### NATIONAL INSURANCE ACT DEVELOPMENTS.

### The New Amendment Act.

Another National Insurance Amendment Act has been placed upon the Statute Book as part of the emergency legislation due to the war. The Bill was introduced in the House of Commons on March 4, and, having passed through all its stages in that Chamber by March 9, it was then considered by the House of Lords and obtained its third reading on the 11th. Thus only a week elapsed from the initial to the final stage, and this speed is in marked contrast to the procedure in the passage not only of the principal Act but also of the previous Amendment Acts. The reason for the greater expedition in the procedure lies in the fact that the measure was claimed to be of a non-contentious character, and, although it did not pass without criticism, it may fairly be said that no serious opposition was evidenced.

### Relief to Approved Societies in Respect of Disabled Soldiers.

The Act consists of three operative sections, the first of which is designed to give effect to the recommendation of the Select Committee of the House of Commons which was appointed to consider the question of pensions and separation allowances to the Forces of the Crown. This recommendation was to the effect that the pension for soldiers who are totally disabled should be 25s. a week, and that this sum should include, and not be in addition to, any disablement benefit which might be receivable under the Insurance Act.

There were two ways in which this recommendation might have been construed. It might, for instance, have been taken to indicate that when the disabled soldier was entitled to disablement benefit of 5s. a week under the National Insurance Act the pension payable would be reduced to £1, thus bringing up the total to 25s., and imposing the burden of an expensive permanent charge on the approved society. The Government have, however, decided upon the other construction—namely, that when the total disablement pension of 25s. a week is granted the approved society shall be relieved of the payment of the disablement benefit.

There are one or two points in the section which should be emphasised and made quite clear. In the first place, the relief to the approved societies is only in respect of men to whom Section 46 of the principal Act applies at the time of their discharge. That is to say, the first section of the new Act only applies to persons in the naval and military service of the Crown. Furthermore, it only applies to such men as come within the foregoing description who are granted pensions for total disablement suffered in consequence of the present war. The amount of the relief to the approved societies is a reduction of 5s. a week from any sickness or disablement benefit to which the insured member may be entitled, so long as he continues in receipt of such pension.

The medical, sanatorium, and maternity benefits are unaffected, and it is to be presumed from the wording of the section that upon recovery from the disablement the member would be reinstated with full rights and privileges.

Seeing that no contributions are payable while a member is in receipt of sickness or disablement benefit—and again, from the wording of the section, it must be supposed that he will be treated for the purposes of the accounts as though he was entitled to benefit—it is difficult to see how the cost of the three remaining benefits is to be met. It is true, of course, that the relief to the societies of 5s. a week in the circumstances mentioned is the chief essential, but it does not seem that the relief is absolutely complete.

### Prompt Settlement Provided.

There is a second clause to the first section of the Act which makes it possible for approved societies to pay the normal benefits—sickness or disablement—until such time as the settlement of the pension is arranged and allows the society to charge any payments which have been made in excess of the reduced liability as deductions from future benefit payments, or, if the Admiralty or Army Council think fit, the excess payments may be repaid to the society out of any arrears of pension that may be due. This is an exceedingly useful provision, as it enables prompt settlement of claims to be made, and it will therefore be of service to the disabled insured person while at the same time it will afford protection to the approved societies.

### No Double Benefit Obtainable.

The second section is of a similar character to the first in that it affords relief to the approved societies, but it is not restricted solely to the Forces of the Crown.

Various additions have been made, owing to the war, to the persons entitled to obtain compensation for injuries or disablement, and the object of the section is to provide that when compensation similar in character to that granted under the Workmen's Compensation Act is obtained, the member shall not be entitled to draw the National Insurance benefit as well. These first two sections are the only ones that directly affect insured persons. They limit the benefit obtainable under the Acts, but only when a corresponding or a larger benefit is obtainable from another source. In consequence, they do not inflict any hardship upon the insured persons immediately affected. The relief they will afford to the approved societies, and thus to the general body of members, is likely to be considerable; and although the charge is merely transferred, it is transferred to the National Exchequer, which in the circumstances is the right and proper source from which the grants of pensions and compensation should be derived.

Simplification of Procedure.

The third section of the Act relates to the procedure whereby the contributions are credited to the approved societies in respect of Navy and Army members. The substituted process will tend to facilitate the accountancy, as the Navy and Army Insurance Fund is dispensed with as an intermediary between the Insurance Commissioners and the approved societies for those in the Forces of the Crown who are members of societies. The question of the revision of the allowance made to approved societies for administrative purposes in respect of Navy and Army members was raised in the discussion of the Bill. This is one of the most important matters which has now to be determined, but as the Insurance Commissioners have power to deal with it, it is to be decided without special legislation.

